# Ipsa senectus morbus est? Approaching the unmet needs in geriatric depression: results from a national survey of medical experts

**DOI:** 10.3389/fpsyt.2025.1583384

**Published:** 2025-10-15

**Authors:** Umberto Albert, Carmine Tomasetti, Raffaele Antonelli Incalzi, Alessandro Cicolin, Alessandro Padovani, Michela Zanetti, Giuseppe Maina

**Affiliations:** ^1^ Department of Medicine, Surgery and Health Sciences, University of Trieste, Trieste, Italy; ^2^ Unità Clinica Operativa (UCO) Clinica Psichiatrica, Azienda Sanitaria Universitaria Integrata Giuliano-Isontina (ASUGI), Trieste, Italy; ^3^ Department of Mental Health of Teramo, Alzheimer Centre of Giulianova, ASL Teramo, Giulianova, TE, Italy; ^4^ Internal Medicine Unit, Fondazione Policlinico Universitario Campus Bio-Medico, Rome, Italy; ^5^ Regional Sleep Medicine Center, Department of Neurosciences Rita Levi Montalcini, University of Turin, Torino, Italy; ^6^ Department of Clinical and Experimental Sciences, Neurology Unit, University of Brescia, Brescia, Italy; ^7^ San Luigi Gonzaga Hospital, University of Turin, Orbassano, Italy; ^8^ Department of Neurosciences “Rita Levi Montalcini”, University of Turin, Orbassano, Italy

**Keywords:** antidepressants, comorbidity, trazodone, psychiatry, neurology, geriatrics, elderly

## Abstract

**Introduction:**

One of the main challenges of “real-world” management of depression is represented by geriatric depression, which is common and under-diagnosed. Depressive disorders represent the leading contributors to mental health-related global burden, and they are often diagnosed in the context of many comorbid disorders, such as cardiovascular disorders, Stroke, Parkinson’s Disease, Major Neurocognitive Disorders and Headache, thus worsening their outcomes. Depression, and above all geriatric depression, is a challenge for “real-world” clinicians, due to the low rates of remission despite the increasing number of antidepressant strategies currently available. Indeed, current antidepressant strategies often fail to achieve acceptable rates of remission. The challenge of diagnosis and treatment of geriatric depression in real world calls for the need of a deeper exploration of its management in clinical practice. This is the purpose of the present cross-sectional survey, aimed at evaluating the clinical approach to late-life depression in a sample of expert physicians working in geriatric settings in Italy.

**Methods:**

Hereafter, we provide responses from 175 geriatrics-working physicians, which were recruited to participate in the survey through their membership in the Italian Society of Geriatrics and Gerontology (SIGG). They were invited to respond to a a 20-items questionnaire which was developed based both on literature review and on the *a priori* knowledge of the subject by the developing team, composed by seven expert physicians in the fields of Psychiatry, Neurology and Geriatrics

**Results and discussion:**

The survey was aimed at delving into the possible unmet needs in the management of geriatric depression according to the sample of physicians surveyed, thus trying to provide useful insights on geriatric depression care.

## Introduction

Depressive disorders represent the leading contributor to mental health-related global burden; according to the Global Burden of Disease study (1), they have been calculated to lead to 5 million years lived with disability (YLD), thus being ranked as the major cause of non-fatal health loss (7.5% of all YLD) (2), with a suicide rate of 700.000 people per year (1). In Italy, patients diagnosed with depressive disorders represent the most prevalent population in charge to mental health services (34.6/10.000 people), with females almost doubly affected than males (43.2 vs 25.4/10.000 people) (3). Based on worldwide epidemiological studies (e.g. the Royal Australian and New Zealand College of Psychiatrists), depressive symptoms, as captured by the main used rating scale (i.e. Patient Health Questionnarie-9 [PHQ9], Kessler Psychological Distress Scale [K10], Depression Anxiety Stress Scales [DASS] and Hamilton Rating Scale for Depression [HAMD]), have been reported in one out of ten primary care patients (4), and they often arise in the context of many other comorbid disorders, such as endocrine dysfunctions, cardiovascular disorders, stroke, Parkinson’s Disease, Major Neurocognitive Disorders (e.g., Alzheimer’s Disease) and headache, thus complicating their progress and treatment, as well as increasing their burden and affecting the outcomes (5, 6). Moreover, according to a recent systematic review and meta-analysis, since depression is significantly associated with chronic medical disorders, this frequent comorbidity, whenever misdiagnosed, may considerably impair the patient’s perception of medical care as well as increase the economic burden (7). Consolidated evidence, indeed, supports the view of envisioning depression as a mind-body disorder, trying as much as possible to tailor the antidepressant treatments on the individual features of the patient, such as physical characteristics, psychological fragilities, comorbidities, pharmacological treatments, and combining pharmacological and psychotherapeutic treatments to maximize their effectiveness (8, 9).

One of the main challenges of “real-world” management of depression is represented by geriatric depression. Indeed, world population is increasingly growing old, and the proportion of elderly people (aged 60 years and over) has been estimated to increase to 1.4 billions by 2030 and to 2.1 billions by 2050 (10). A recent systematic review and meta-analysis estimated a 28.4% global prevalence of depression in older people (11). Several unmet needs have been associated to the management of geriatric depression: misdiagnosis, underdiagnosis, management of comorbidities, difficulties in choosing safe and effective treatments. All of them may potentially determine a poor prognosis. Depression in older age, indeed, has been associated to worsening of physical and cognitive functions (12), as well as to adverse outcomes due to chronic diseases multimorbidity (13). When approaching depression in elderly patients, physicians often find themselves dealing with a complex condition characterized by suffering, abandonment, and generally compromised functioning. Late-life depression is often diagnosed in the context of multiple comorbidities, both medical and neurological. In fact, cardiovascular and cerebrovascular diseases, thyroid dysfunctions, adrenocortical disorders, diabetes, vitamin B12 and folic acid deficiency, as well as malnutrition, represent the most common medical conditions associated with geriatric depression (14). Also, multiple treatments, which are often used to manage these conditions in elderly people (e.g. antihypertensive medications) may contribute to exacerbate, or to develop, depressive symptoms (15). Last, but not least in importance, poor psychosocial conditions, such as low economic status, abandonment, isolation and relocation, and senile decrepitude (i.e. the loss of abilities and pleasure), which often come with people in the late stages of their lives, may trigger depressive symptoms or worsen pre-existing depression (15). Moreover, depression has been reported in 30% of people diagnosed with vascular dementia or Alzheimer’s Disease, and in 40% of patients diagnosed with dementia associated to Parkinson’s or Huntington’s diseases (16). Thus, finding the appropriate treatment combination for elderly people with depression, trying to avoid as much as possible pharmacological interactions, side effects, and to maximize the effectiveness, may represent a hard task for physicians (17, 18).

The challenge of diagnosis and treatment of geriatric depression in real world calls for the need of a deeper exploration of its management in clinical practice. In the wake of this worldwide wave of readjustment of the real-world clinical practice to the challenges of depression in the elderly (19, 20), the purpose of the present cross-sectional survey is to evaluate the clinical approach to late-life depression in a sample of expert physicians working in geriatric settings in Italy, thus trying to provide useful insights on geriatric depression care.

## Methods

### Study design

The present cross-sectional survey-based study follows the STROBE (Strengthening the Reporting of Observational studies in Epidemiology) guidelines, matching the appropriate checklist (21).

### Setting and participants

Geriatrics-working physicians were recruited to participate in this survey through the Italian Society of Geriatrics and Gerontology and were only eligible if they had active membership. The Italian Society of Gerontology and Geriatrics (SIGG – Società Italiana di Gerontologia e Geriatria) is the national scientific society that brings together professionals involved in the care, study, and management of aging and the elderly. Founded in 1950, SIGG promotes clinical research, continuous medical education, and the development of national guidelines. Its activities include: an annual national congress and multiple regional events; the participation in national consensus and clinical recommendations; the promotion of in-service training through workshops, webinars, and CME-accredited courses; the coordination of thematic working groups (e.g., neurodegeneration, multimorbidity, pharmacology, frailty). As of 2024, SIGG counts more than 2100 members across Italy, with a majority being medical doctors specialized in geriatrics, working in hospitals, universities, long-term care facilities, and territorial health services.

At the time of the survey, SIGG had 868 active members, and a total of 175 physicians responded to the survey (confidence level = 95%, confidence interval = 6.62%). Participant recruitment and data collection occurred between September and November 2023, with individual emails containing the survey link at launch, and a reminder after four weeks.

### Questionnaire and variables studied

A 20-items questionnaire was developed based both on literature review (22–25) and on the *a priori* knowledge of the subject by the developing team, by using the Nominal Group Technique (26). The team was composed by seven expert physicians in the fields of Psychiatry, Neurology and Geriatrics, which firstly debated the issue of unmet needs in the treatment of geriatric depression based on their own experience, and then reviewed the literature to generate ideas in order to develop and implement the questionnaire. The literature review provided insights on the following themes: prevalence and clinical impact of geriatric depression; unmet needs in approaching and treating geriatric depression; clinical practices in managing geriatric depression; therapeutic approaches to geriatric depression; strategies and safety of pharmacological treatments in geriatric depression. The experts were asked to create specific questions on each theme. According to the NGT, questions were ranked using a computerized tool to calculate percentage of preferences by the experts, then debated again and finally used to create the final questionnaire. The survey assessed demographic, employment and clinical practice characteristics, as well as the basic knowledge in the field of geriatric depression diagnosis and treatment. Then, it delved into the possible unmet needs in the management of geriatric depression according to the sample of physicians surveyed. The 20-item questionnaire is entirely shown in **Supplementary Material**.

### Data curation and statistical analysis

The questionnaire allowed one single answer, and no open-text responses were permitted. Questionnaire responses were analyzed in terms of simple distribution (percentage) of the answers, with a descriptive analysis, and reported in figures. Data were stored on secure servers for revision and possible future further analyses.

## Results

As above mentioned, a total of 175 geriatrics-working physicians responded to the survey, with an approximate response rate of 20%, as compared to the total amount of SIGG active registered physicians. The following results are presented showing both the raw number of respondents on the total and the calculated respective percentage. Both results and figures have been organized in thematic clusters resembling those debated by the experts (see Methods).

### Demographics of surveyed health professionals

Demographic data of the sample have been summarized in **Table 1**. 42.3% of respondents were under 40 years old, and the majority (64%) were females. Mostly, they declared a clinical experience between 6 and 25 years in the field of geriatrics, and, indeed, the majority were specialized in Geriatrics (94.28%). The most part was working in the Public National Health System (59.4%), preferentially in acute cases-dealing wards (38.85%). The geographic distribution favored North Italian regions (65%).

**Table 1:** Demographic characteristics of the sample of SIGG surveyed physicians.

Total Sample (n)	175
Age	
30-35	50 (28.6%)
36-40	24 (13.7%)
41-45	14 (8%)
46-50	16 (9.1%)
51-55	25 (14.3%)
56-60	12 (6.9%)
61-65	20 (11.4%)
66-70	10 (5.7%)
>70	4 (2.3%)
Gender	
Male	63 (36%)
Female	112 (64%)
Years of experience	
1-5	47 (26.9%)
6-10	19 (10.9%)
11-15	20 (11.4%)
16-20	23 (13%)
21-25	24 (13.7%)
26-30	8 (4.6%)
31-35	12 (6.9%)
36-40	10 (5.7%)
41-45	8 (4.6%)
46-50	4 (2.3%)
Specialization	
Geriatrics	165 (94.3%)
Internal medicine	8 (4.6%)
General medicine	2 (1.1%)
Work structure	
Public structure	104 (59.4%)
Private structure	26 (14.9%)
University hospital	40 (22.9%)
Third sector organisation	2 (1.1%)
Freelancer	3 (1.7%)
Working environment	
RSA	25 (14.3%)
Acute care unit	68 (38.8%)
Rehabilitation ward	18 (10.3%)
Long-term care	6 (3.4%)
Home care	19 (10.9%)
Outpatient clinic	33 (18.9%)
Emergency room	4 (2.3%)
Research institute	2 (1.1%)
Geographical distribution	
Northern Italy	114 (65.1%)
Central Italy	33 (18.9%)
Southern Italy	28 (16%)

### Prevalence and clinical impact of geriatric depression

**Figure 1:**
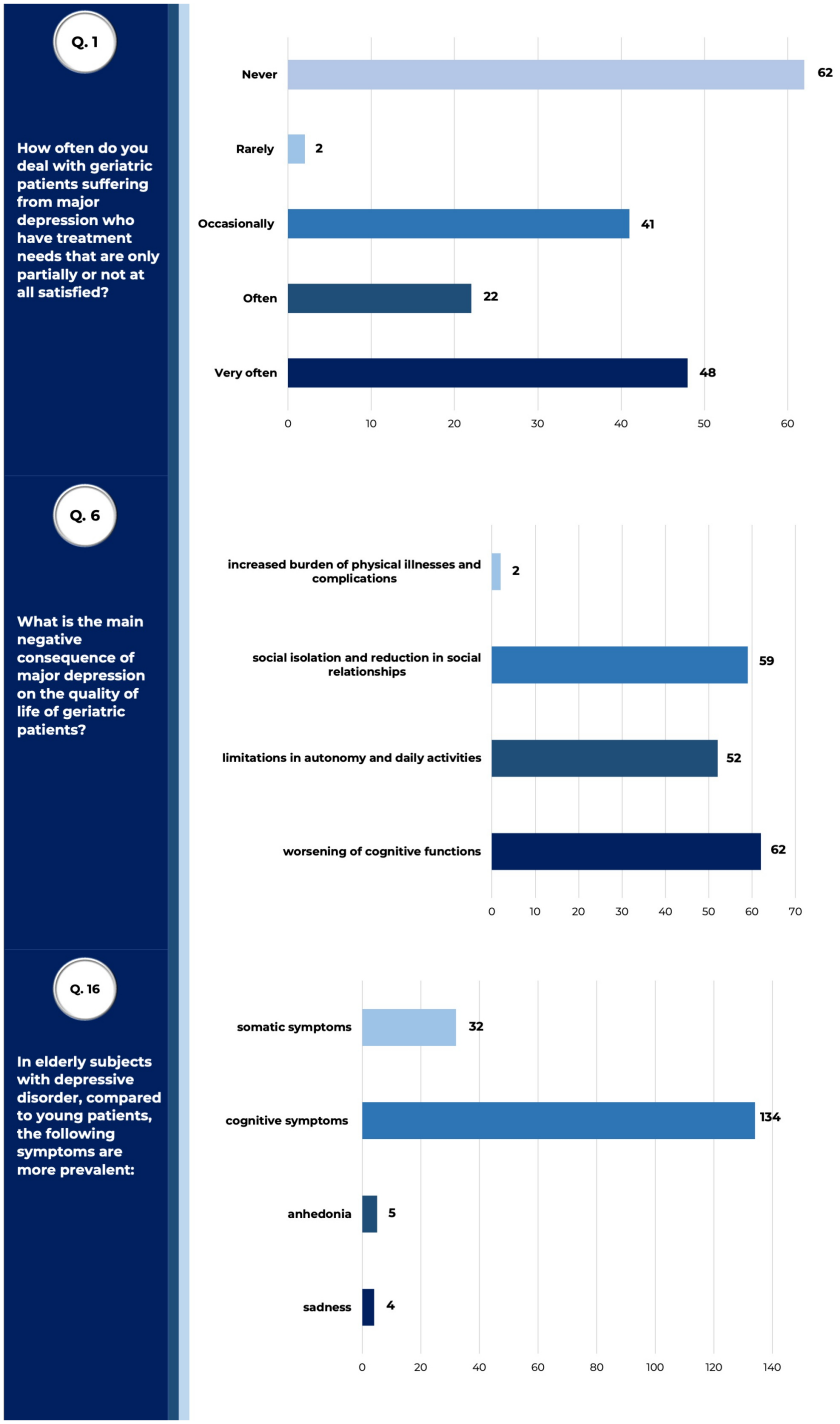
Prevalence and clinical impact of geriatric depression. Answers to queries 1, 6, and 16 of the questionnaire submitted to SIGG members, relative to results described. Raw data are reported (tot. surveyed physicians =175).

Forty percent of physicians declared that they dealt often (22/175), or very often (48/175), with elderly patients diagnosed with depression showing unsatisfied care needs. Indeed, the responders strongly agreed (76.6% - 134/175) that elderly patients with depression preferentially manifest cognitive symptoms, as compared to depressed adult patients (**Figure 1**). Physicians also agreed that the main consequences of geriatric depression may be represented by a worsening in cognitive functioning (35.4% - 62/175), social withdrawal (33.7% - 59/175) and daily autonomies limitations (29.7% - 52/175).

### Unmet needs in approaching and treating geriatric depression

**Figure 2:**
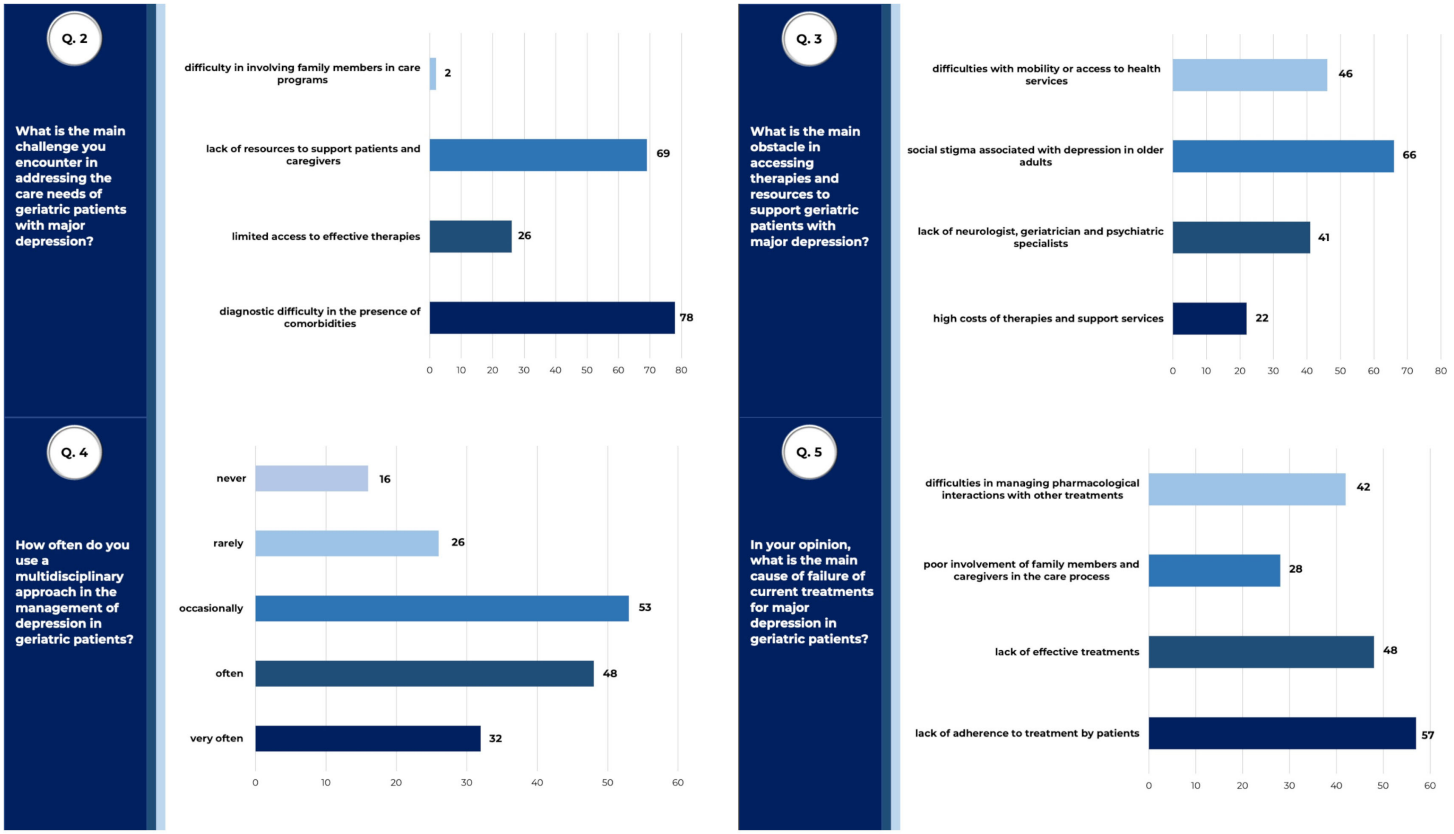
Unmet needs in approaching and treating geriatric depression. Answers to queries 2, 3, 4 and 5 of the questionnaire submitted to SIGG members, relative to results described. Raw data are reported (tot. surveyed physicians =175).

The main challenges in addressing care needs of geriatric patients were represented by medical comorbidities (44.6% - 78/175) and by scarceness of patients’ or caregivers’ support (39.4% - 69/175). The respondents agreed at 37.71% (66/175) that the main obstacle to care was the stigma associated to depression in elderly, followed by physical barriers making it difficult to access health services (26.3% - 46/175). Non-compliance to treatments (32.6% - 57/175), followed by lack of effective treatments (27.4% - 48/175) and difficulties in managing pharmacological interactions (24% - 42/175) were considered the main causes of therapeutic failure. Anyway, due to multiple comorbidities and difficulties, most part of physicians declared to take advantage of a multidisciplinary approach to depression treatment in geriatric patients (**Figure 2**).

### Managing geriatric depression in clinical practice

**Figure 3:**
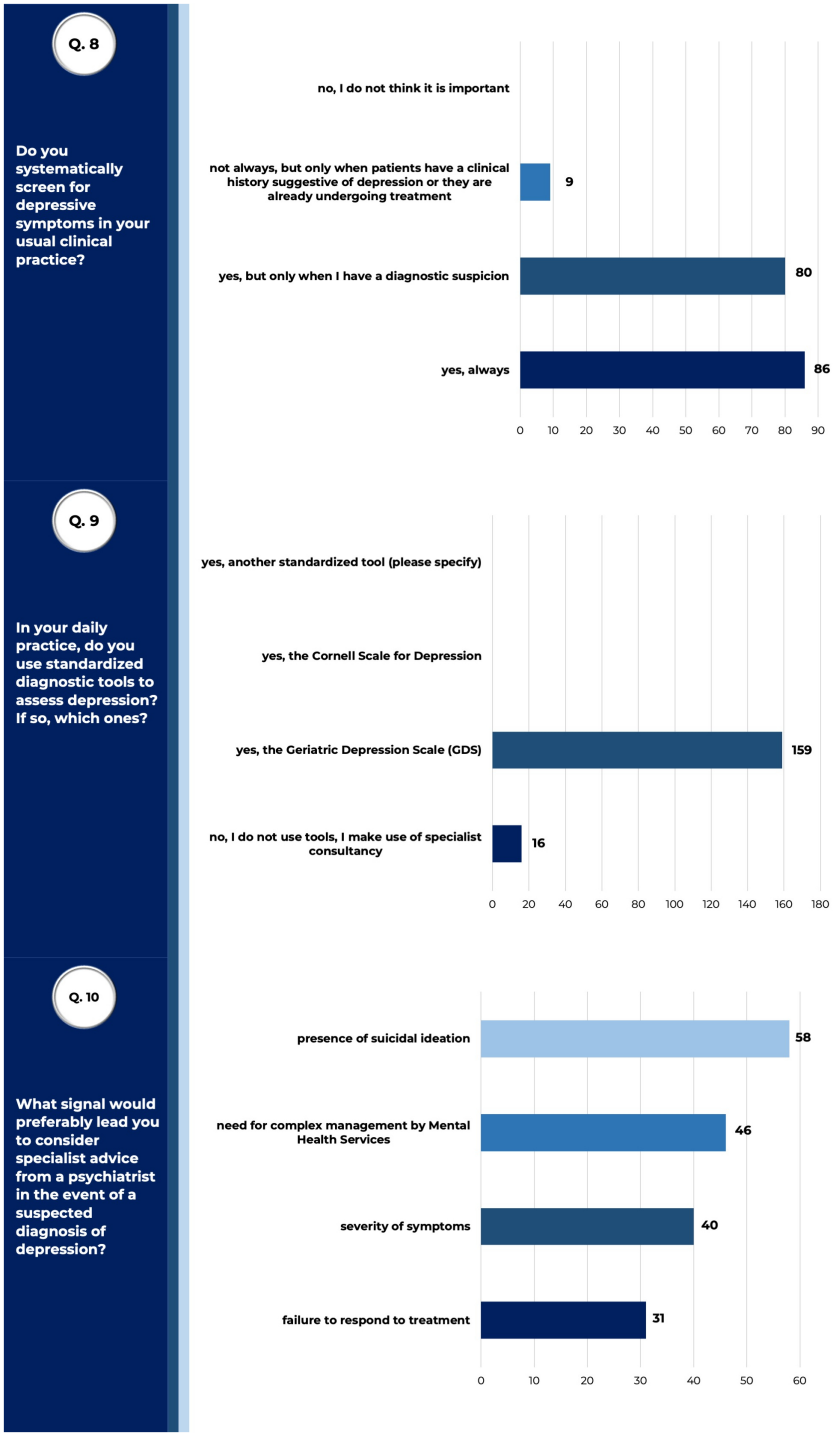
Managing geriatric depression in clinical practice. Answers to queries 8, 9 and 10 of the questionnaire submitted to SIGG members, relative to results described. Raw data are reported (tot. surveyed physicians =175).

Almost all respondents declared to systematically screen geriatric patients for depressive symptoms (both only when having a diagnostic suspicion 80/175 and always 86/175), mostly by using the Geriatric Depression Scale (GDS) (159/175) as a standardized tool (27) (**Figure 3**). GDS is a 30-items questionnaire (a 15-items short version is also available) first developed by Yesavage (28), and extensively validated, in which patients are asked to respond to yes or no questions about how they felt over the past week: 0–9 scoring is considered normal, 10–19 suggests mild depression, 20–30 severe depression.

Respondents declared to avail themselves of a psychiatric consultation mainly in the event of suicidal ideation shown by patients (33.14% - 58/175), or when patients need a complex undertaking by mental health services (26.3% - 46/175).

### Therapeutic approaches to geriatric depression

**Figure 4:**
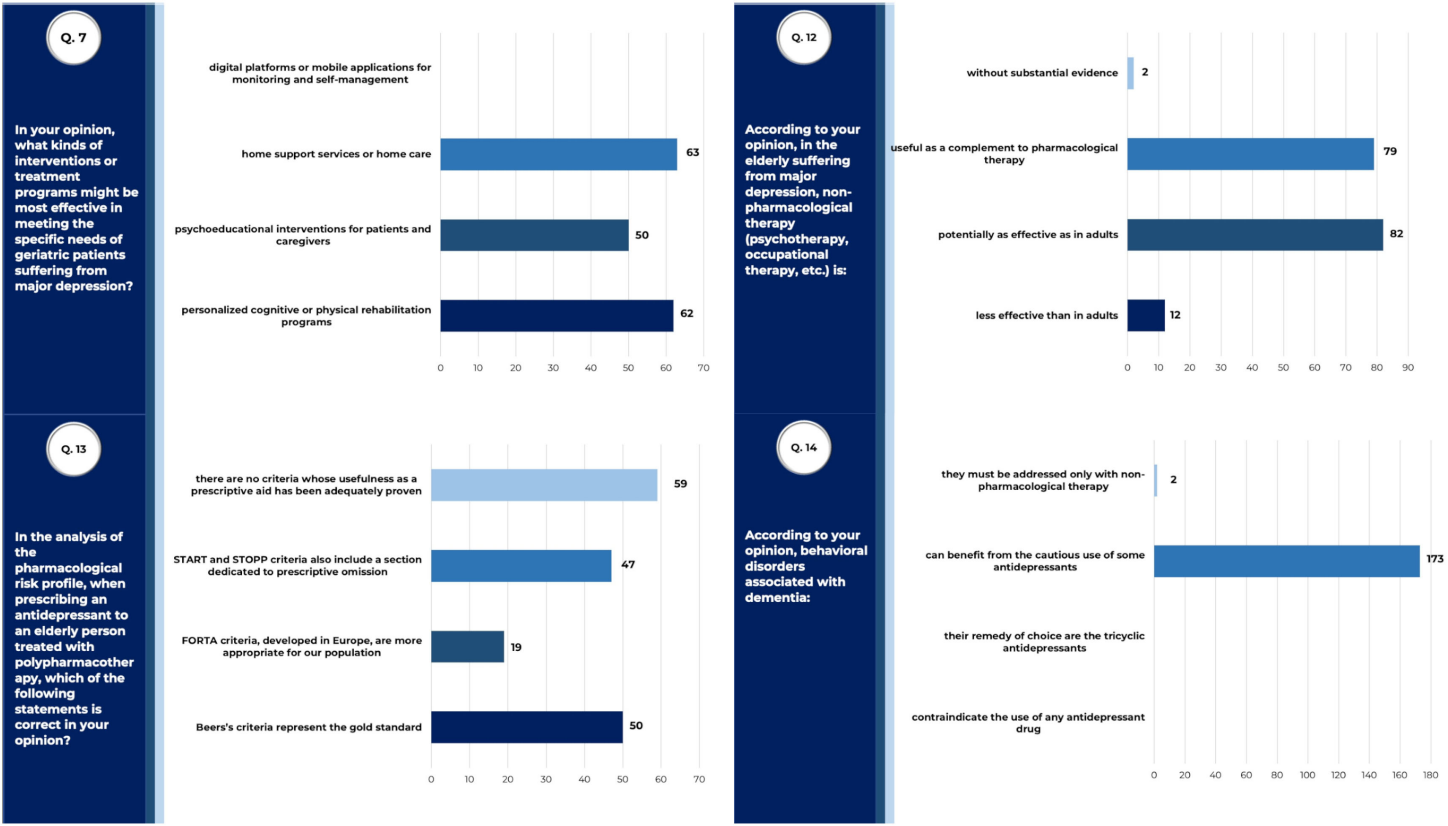
Therapeutic approaches to geriatric depression. Answers to queries 7, 12, 13 and 14 of the questionnaire submitted to SIGG members, relative to results described. Raw data are reported (tot. surveyed physicians =175).

When choosing a therapeutic strategy, most of surveyed physicians (45.14% - 79/175) agreed that non-pharmacological treatments should be proposed as a completion of pharmacological treatments (**Figure 4**), due to their well-known combined efficacy, comparable to that in adult population (46.85% - 82/175). Responders agreed also that patients may benefit from domestic support services (36% - 63/175), individualized cognitive and physical rehabilitation programs (35.4% - 62/175), as well as psychoeducational intervention for both patients and caregivers (28.6% - 50/175). Regarding the use of pharmacological treatments, the responders almost equally chose the use of specific guidelines of treatment, such as the Beers’s criteria, the Screening Tool of Older Person’s Prescriptions (STOPP) criteria, the Screening Tool to Alert doctors to the Right Treatment (START) criteria, and the Fit fOR The Aged (FORTA) list (29) (**Figure 4**). All these criteria have been developed to avoid polypharmacy and inappropriate prescriptions in older patients, which are commonly considered as risk factors for adverse drug reactions, as well as a common cause of worse clinical outcomes in elderly (30). The Beers’s criteria were first developed in 1991 as a drug-oriented listing approach (DOLA) focusing on listing inappropriate medications for older patients (31). Although being the most widely used criteria, some limitations have been found, such as that they do not account for prescribing omissions (32). Thus STOPP/START criteria were developed as an explicit set of criteria based on the physiological system (e.g. cardiovascular system, central nervous system) impacted by drugs, in order to attempt inappropriate prescriptions, omissions and interactions. STOPP/START criteria were validated using a Delphi consensus methodology and randomized controlled trials (RCTs) demonstrated a significant improvement in appropriateness and reduced prescribing omissions (33). FORTA criteria represent a patient-in-focus listing approach (PILA), which are constantly updated and revised. Their prerequisite is a complete approach to older patient, with an analysis of the best fitting drug prescription according to diagnosis, severity, life expectancy, functional status, and patient wishes, which has been demonstrated useful to optimize treatment strategies in the elderly (34).

Finally, almost all the surveyed physicians agreed that antidepressant drugs could be used cautiously to treat behavioral disorders in geriatric patients (98.85% - 173/175).

### Strategies and safety of pharmacological treatments in geriatric depression

**Figure 5:**
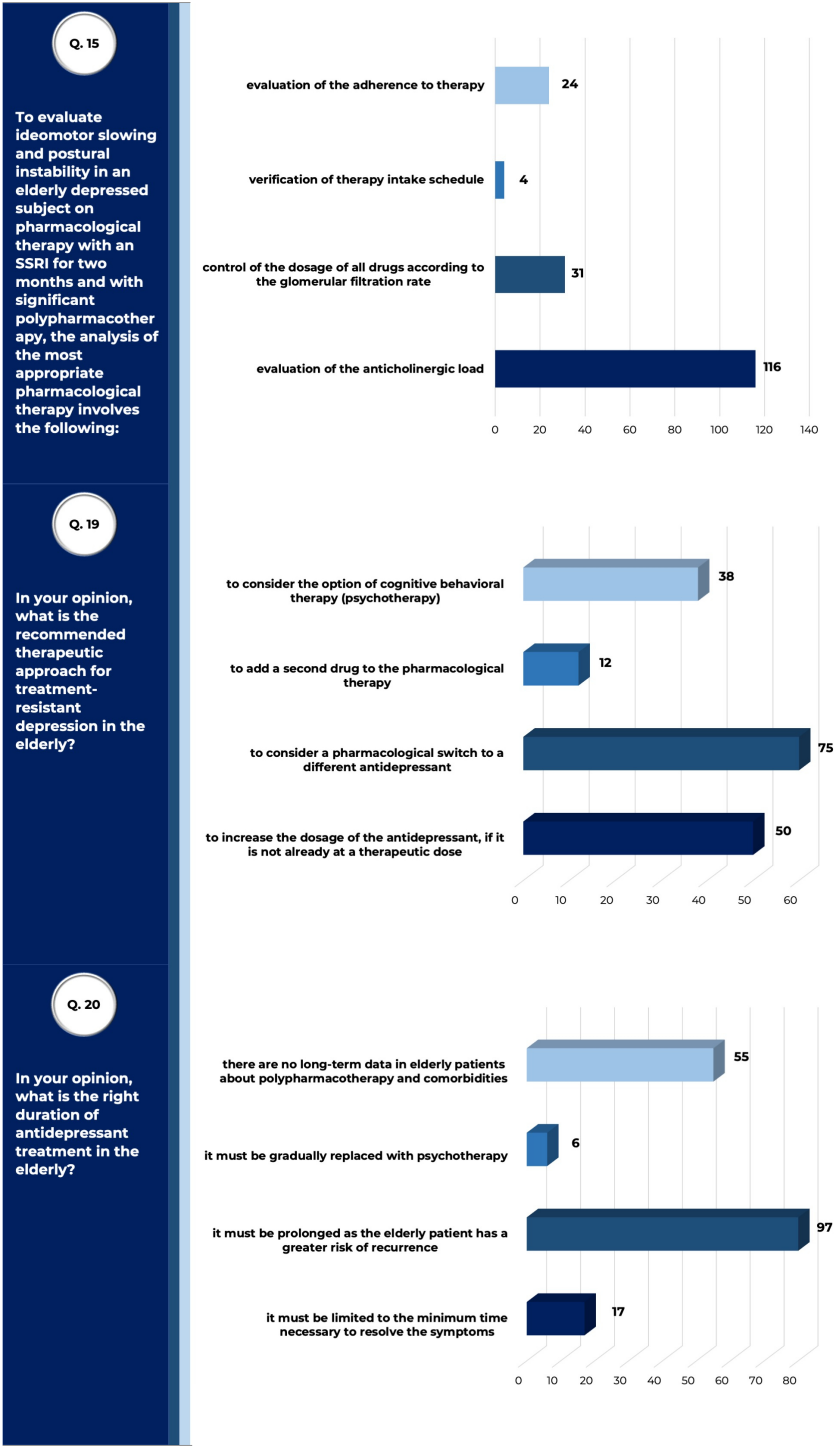
Strategies and safety of pharmacological treatments in geriatric depression. Answers to queries 15, 19 and 20 of the questionnaire submitted to SIGG members, relative to results described. Raw data are reported (tot. surveyed physicians =175).

Most surveyed physicians (66.3% - 116/175) agreed that the so-called anticholinergic drug load (35) should be considered when prescribing antidepressants in poly-treated elderly patients (**Figure 5**). A significant number of responders also believed that, in case of treatment-resistant geriatric depression, it should be firstly evaluated an increase in the dosage of the antidepressant, if not already at therapeutic dose (28.57% - 50/175), as well as a switch to a different antidepressant drug should be considered (42.85% - 75/175); then, a combined psychotherapeutic (cognitive-behavioral therapy) approach should be initiated (21.7% - 38/175). The duration of antidepressant pharmacological treatment should be prolonged, due to the higher risk of relapses in older patients as compared to adults (55.4% - 97/175).

## Discussion

To the best of our knowledge, this is the first survey on an Italian sample to specifically analyze the opinion of expert physicians on geriatric depression. The experts of SIGG were asked to give their opinion on diverse clinical problems and unmet needs regarding their experience with patients suffering from geriatric depression. 175 physicians responded to the survey. This might be considered a small sample, but according to demographics it seems representative of the actual context of geriatrics-working in Italy. Indeed, SIGG reflects a multidisciplinary and cross-setting community, making it a valuable context for exploring professional perspectives on geriatric depression. The over-representation of respondents from acute care settings, public institutions, and Northern Italy can be partly explained by the real-world distribution of geriatric services and academic centers in Italy. First, geriatric medicine in Italy is primarily integrated into the public health system, particularly through public hospitals and university-affiliated centers, which naturally form the backbone of geriatric care and research. This explains the predominance (~50%) of respondents from public structures. Second, the Northern regions of Italy historically have a higher concentration of geriatric departments, teaching hospitals, and SIGG-affiliated professionals, reflecting the regional organization of healthcare and a longer-standing tradition in geriatric education and research. This aligns with the higher participation rate from those areas. Finally, acute care settings often represent the first point of contact for complex elderly patients with depression, multimorbidity, and functional decline. Professionals working in these settings may have been more motivated to participate in a survey on unmet needs in geriatric depression, thus contributing to the ~40% representation.

According to the presented results, Italian geriatrics-working physicians very often deal with older patients diagnosed with depression, which show unsatisfied care needs. In particular, the most part of respondents agreed that comorbidities in older patients suffering from depression may impair a correct treatment strategy, because of the actual lack of effective treatments and the difficulties in managing pharmacological interactions and side effects, which also lead to scarce adherence to treatments, together with the poor involvement of caregivers in the care process. Several studies, indeed, demonstrated that depression is often associated to comorbid medical illnesses in elderly patients, these comorbidities increasing synergistically the odds of disability in basic and instrumental activities of daily living, as well as interactively worsening the quality of life of affected patients (36). Therefore, a combined treatment taking into account the multimorbidity may be helpful to increase effectiveness of treatments in elderly depressed patients (37) Recently, some authors have envisioned the possibility of review “old” psychopharmacotherapies, such as trazodone, in the light of their safe and effective use in older multimorbid patients, based on the scarce pharmacodynamic and pharmacokinetic interactions, the rapidity of actions, and the efficacy in complex cotreatment contexts (22).

According to this view, a multidisciplinary approach to treat depression in elderly population has been increasingly considered a better strategy as compared to non-integrated protocols, since it emphasizes the collaboration amongst different professionals (i.e. general practitioners, psychiatrists, psychologists, neurologists, geriatricians, nurses, and others) and provides a more feasible success against the multiple challenges of older people depression (38). This vision is completely embraced by physicians that responded to this survey, which agreed that a multidisciplinary approach to the treatment of depression in older people may represent and advantage in overcoming difficulties such as the lack of compliance to therapy, the choice of effective and safe pharmacological treatments, as well as the management of polypharmacy-related interactions.

Responders also agreed that stigma may represent a main obstacle to a correct treatment approach to older people depression. Indeed, negative attitudes toward older people, such as prejudices, ageism, stereotypes, underestimation, may significantly impair the right complex vision of depression in the elderly, thus discriminating the provision of combined physical and mental health services to these patients (39). Specific multidisciplinary programs, as well as aimed research about the issue, may help to avoid the stigmatization of care for older people with comorbid physical problems (40). Home support and psychoeducational programs for both patients and caregivers may also help to avoid stigmatic attributions by caregivers, which impair the effectiveness of treatments and, in turn, favor the onset of caregivers’ stress-related burden (41).

A large percentage of responders of this survey agreed on the use of validated tool, such as the GDS to correctly diagnose depression in older people. Indeed, several studies have demonstrated the high sensitivity and specificity of GDS for detecting depression in the elderly (42). Standardized evaluation scales represent useful tools also to avoid misdiagnosis of depression in older people, which are often underdiagnosed (and then undertreated) due to the multiple comorbidities which may mask depressive symptoms, or even to stigmatic attribution such as that depression may be part of growing old (43). Moreover, consultation with specialized physicians, such as psychiatrists, may improve the effectiveness of diagnosis and treatment management, particularly in case of complex situations which should be taken in charge by mental health services, such as the emerging of suicidal thoughts or intentions (44). Indeed, untreated depression, due to misdiagnosis or underestimation, has been demonstrated to worsen the quality of life of older patients, as well as to increase the odds of developing a neurocognitive disorder (45). To meet these increasing needs in geriatric depression, in some countries a specialized figure of Geriatric Psychiatrist is emerging (46).

The majority of surveyed physicians claimed that pharmacological treatments are essential to avoid the progression of depressive symptoms in older people, and to prevent cognitive decline in these patients. However, pharmacological treatments should be combined with non-pharmacological interventions to maximize the effectiveness, in the light of the emergent multidisciplinary and team approach to geriatric depression. This is in agreement with consistent evidence on the favorable effect of non-pharmacological interventions in support of pharmacological treatments in elderly depressed patients to improve the outcomes, to reduce the progression of cognitive deterioration, and also to reduce dosages of the drugs used, as well as to avoid multiple psychopharmacological combinations (with a heightened risk of side effects) (47–49). Although antidepressants are essential for the treatment, most of the responders agreed that these drugs should be prescribed to older people following validated criteria to reduce over-prescription, side effects, and to optimize the effectiveness of treatment. Beers, START, STOPP, FORTA criteria have been all developed to improve pharmacotherapy in older patients, and have been demonstrated to be useful to maximize treatments efficacy and minimize unwanted side effects (17, 18, 23, 50–52). Moreover, when prescribing antidepressant drugs to older people, their anticholinergic properties should be considered. Indeed, many drugs used by older people to treat common conditions (i.e. allergy, overactive bladder, anxiety) have anticholinergic properties, which have been directly correlated to worsening in cognition, drowsiness, falls and constipation (53). The so-called anticholinergic burden has been demonstrated to worsen the daily abilities of elderly depressed patients, as well as to promote their cognitive deterioration (54–57). Thus, when prescribed, antidepressants possibly lacking anticholinergic properties should be preferred. According to this view, some antidepressant drugs with anxiolytic/antidepressant properties, such as trazodone, have been envisioned as first-choice treatment in behavioral manifestations of older depressed patients (58), due to the lack of anticholinergic effects, the primary effects on sleep architecture (59), and the important pro-cognitive and anti-deteriorative effects (60).

Citalopram, escitalopram and sertraline have also been considered to show a safe profile in the treatment of depression in the elderly, due to their low propensity to drug-to-drug interactions (61). Recently, also vortioxetine has been demonstrated to show beneficial effects and a safe profile comparable to SSRIs, although without clear tolerability advantages over the older drugs (62). However, given the established increase in adverse effects when antidepressants are prescribed to older patients (63, 64), the surveyed physicians, supported by literature data, agreed on a long-lasting duration of antidepressant treatment in older people, both to optimize treatment and to avoid relapses (23), as well as on the support of cognitive-behavioral therapy in partially effective treatments, in order to maximize the efficacy (65).

## Conclusions

Geriatric depression may represent a hard challenge for physicians, due to the high rates of misdiagnosis and undertreatment, to the difficult management of comorbidities, as well as to the development of an effective treatment strategy. The present survey represents a first attempt to collect the experiences of geriatrics-expert physicians in order to provide useful insights on the unmet needs in the treatment of late-life depression. Our cross-sectional study aims at being part of the upcoming researches and reviews exploring the possible approaches to the challenges posed by the ever-increasing diffusion of depression in older population, clearly due to world people aging (66–71). Thus, we hope that the present observations may serve as stimulus to further research and debate on the treatment of elderly depression, to develop better and better strategies which may avoid underdiagnosis and help to bypass stigmatization and to improve the outcomes of older patients.

## Data Availability

The original contributions presented in the study are included in the article/**Supplementary Material**. Further inquiries can be directed to the corresponding author.
