# Supplementary material for: Ipsa senectus morbus est? Approaching the unmet needs in geriatric depression: results from a national survey of medical experts
Source: Front Psychiatry. 2025 Oct 15;16:1583384. doi: 10.3389/fpsyt.2025.1583384 (PMC12569647; doi:10.3389/fpsyt.2025.1583384)
Supplement: Supplementary file 1 [file DataSheet1.docx]

**Supplementary Material 1**

**Questionnaire submitted to SIGG members**

1. How often do you deal with geriatric patients suffering from major depression who have treatment needs that are only partially or not at all satisfied?
   1. very often
   2. often
   3. occasionally
   4. rarely
   5. never
2. What is the main challenge you encounter in addressing the care needs of geriatric patients with major depression?
   1. diagnostic difficulty in the presence of comorbidities
   2. limited access to effective therapies
   3. lack of resources to support patients and caregivers
   4. difficulty in involving family members in care programs
3. What is the main obstacle in accessing therapies and resources to support geriatric patients with major depression?
   1. high costs of therapies and support services
   2. lack of neurologist, geriatrician and psychiatric specialists
   3. social stigma associated with depression in older adults
   4. difficulties with mobility or access to health services
4. How often do you use a multidisciplinary approach in the management of depression in geriatric patients?
   1. very often
   2. often
   3. occasionally
   4. rarely
   5. never
5. In your opinion, what is the main cause of failure of current treatments for major depression in geriatric patients?
   1. lack of adherence to treatment by patients
   2. lack of effective treatments
   3. poor involvement of family members and caregivers in the care process
   4. difficulties in managing pharmacological interactions with other treatments
6. What is the main negative consequence of major depression on the quality of life of geriatric patients?
   1. worsening of cognitive functions
   2. limitations in autonomy and daily activities
   3. social isolation and reduction in social relationships
   4. increased burden of physical illnesses and complications
7. In your opinion, what kinds of interventions or treatment programs might be most effective in meeting the specific needs of geriatric patients suffering from major depression?
   1. personalized cognitive or physical rehabilitation programs
   2. psychoeducational interventions for patients and caregivers
   3. home support services or home care
   4. digital platforms or mobile applications for monitoring and self-management
8. Do you systematically screen for depressive symptoms in your usual clinical practice?
   1. yes, always
   2. yes, but only when I have a diagnostic suspicion
   3. not always, but only when patients have a clinical history suggestive of depression or they are already undergoing treatment
   4. no, I do not think it is important
9. In your daily practice, do you use standardized diagnostic tools to assess depression? If so, which ones?
   1. no, I do not use tools, I make use of specialist consultancy
   2. yes, the Geriatric Depression Scale (GDS)
   3. yes, the Cornell Scale for Depression
   4. yes, another standardized tool (please specify)
10. What signal would preferably lead you to consider specialist advice from a psychiatrist in the event of a suspected diagnosis of depression?
    1. failure to respond to treatment
    2. severity of symptoms
    3. need for complex management by Mental Health Services
    4. presence of suicidal ideation
11. Based on your experience, which of the following drugs is best able to alleviate anorexia in depression?
    1. sertraline
    2. escitalopram
    3. venlafaxine
    4. mirtazapine
12. According to your opinion, in the elderly suffering from major depression, non-pharmacological therapy (psychotherapy, occupational therapy, etc.) is:
    1. less effective than in adults
    2. potentially as effective as in adults
    3. useful as a complement to pharmacological therapy
    4. without substantial evidence
13. In the analysis of the pharmacological risk profile, when prescribing an antidepressant to an elderly person treated with polypharmacotherapy, which of the following statements is correct in your opinion?
    1. Beers’s criteria represent the gold standard
    2. FORTA criteria, developed in Europe, are more appropriate for our population
    3. START and STOPP criteria also include a section dedicated to prescriptive omission
    4. there are no criteria whose usefulness as a prescriptive aid has been adequately proven
14. According to your opinion, behavioral disorders associated with dementia:
    1. contraindicate the use of any antidepressant drug
    2. their remedy of choice are the tricyclic antidepressants
    3. can benefit from the cautious use of some antidepressants
    4. they must be addressed only with non-pharmacological therapy
15. To evaluate ideomotor slowing and postural instability in an elderly depressed subject on pharmacological therapy with an SSRI for two months and with significant polypharmacotherapy, the analysis of the most appropriate pharmacological therapy involves the following:
    1. evaluation of the anticholinergic load
    2. control of the dosage of all drugs according to the glomerular filtration rate
    3. verification of therapy intake schedule
    4. evaluation of the adherence to therapy
16. In elderly subjects with depressive disorder, compared to young patients, the following symptoms are more prevalent:
    1. sadness
    2. anhedonia
    3. cognitive symptoms
    4. somatic symptoms
17. According to your opinion, the prevalence of depressive disorder in the elderly is lower among patients with:
    1. Parkinson’s disease
    2. stroke
    3. myocardial infarction
    4. diabetes
18. Which one of the following statements is most likely when assessing a depressed elderly individual who has experienced an increase in blood pressure after starting venlafaxine therapy?
    1. there is no causal link
    2. the causal link is very probable
    3. we have no elements to decide on a possible relationship
    4. drug-drug interactions must be checked
19. In your opinion, what is the recommended therapeutic approach for treatment-resistant depression in the elderly?
    1. to increase the dosage of the antidepressant, if it is not already at a therapeutic dose
    2. to consider a pharmacological switch to a different antidepressant
    3. to add a second drug to the pharmacological therapy
    4. to consider the option of cognitive behavioral therapy (psychotherapy)
20. In your opinion, what is the right duration of antidepressant treatment in the elderly?
    1. it must be limited to the minimum time necessary to resolve the symptoms
    2. it must be prolonged as the elderly patient has a greater risk of recurrence
    3. it must be gradually replaced with psychotherapy
    4. there are no long-term data in elderly patients about polypharmacotherapy and comorbidities
